# Ex Vivo Antioxidant Capacities of Fruit and Vegetable Juices. Potential In Vivo Extrapolation

**DOI:** 10.3390/antiox10050770

**Published:** 2021-05-12

**Authors:** Alexis Matute, Jessica Tabart, Jean-Paul Cheramy-Bien, Claire Kevers, Jacques Dommes, Jean-Olivier Defraigne, Joël Pincemail

**Affiliations:** 1 Laboratory of Plant Molecular Biology and Biotechnology, UR InBios-Phytosystems, University of Liège, Sart Tilman, 4000 Liège, Belgium; afmmatute@student.uliege.be (A.M.); jessica.tabart@alumni.uliege.be (J.T.); c.kevers@uliege.be (C.K.); j.dommes@uliege.be (J.D.); 2 Department of Cardiovascular Surgery, CREDEC and Platform Nutrition Antioxydante et Santé, CHU and University of Liège, Sart Tilman, 4000 Liège, Belgium; jp.cheramy-bien@chuliege.be (J.-P.C.-B.); jo.defraigne@chuliege.be (J.-O.D.)

**Keywords:** fruit and vegetable juices, polyphenols, ORAC assay, ex vivo inhibition of superoxide anion, chemiluminescence assay

## Abstract

Background: In support of claims that their products have antioxidant properties, the food industry and dietary supplement manufacturers rely solely on the in vitro determination of the ORAC (oxygen radical antioxidant capacity) value, despite its acknowledged lack of any in vivo relevance. It thus appears necessary to use tests exploiting biological materials (blood, white blood cells) capable of producing physiological free radicals, in order to evaluate more adequately the antioxidant capacities of foods such as fruit and vegetable juices. Materials: Two approaches to assessing the antioxidant capacities of 21 commercial fruit and vegetable juices were compared: the ORAC assay and the “PMA–whole blood assay,” which uses whole blood stimulated by phorbol myristate acetate to produce the superoxide anion. We described in another paper the total polyphenol contents (TPCs) and individual phenolic compound contents of all the juices were investigated. Results: Ranking of the juices from highest to lowest antioxidant capacity differed considerably according to the test used, so there was no correlation (*r* = 0.33, *p* = 0.13) between the two assays when considering all juices. Although the results of the ORAC assay correlated positively with TPC (*r* = 0.50, *p* = 0.02), a much stronger correlation (*r* = 0.70, *p* = 0.004) emerged between TPC and % superoxide anion inhibition. In the PMA–whole blood assay, peonidin-3-*O*-glucoside, epigallocatechin gallate, catechin, and quercetin present in juices were found to inhibit superoxide anion production at concentrations below 1 µM, with a strong positive correlation. Conclusions: Associated with the determination of total and individual phenolic compounds contained in fruit and vegetable juices, the PMA–whole blood assay appears better than the ORAC assay for evaluating juice antioxidant capacity.

## 1. Introduction

Many papers have highlighted the beneficial role of polyphenols in preventing several human pathologies (cardiovascular and neurodegenerative disorders, cancer, diabetes [1,2,3,4,5]) where increased oxidative stress and inflammation are observed. The beneficial effects of polyphenols are mainly attributed to anti-inflammatory, antioxidant, and anti-platelet-aggregation properties; stimulation of gene-encoding antioxidant enzymes (hormetic effect); improvement in endothelial function leading to good regulation of blood arterial pressure; a decreased glycemic index; epigenetic regulation; and telomere length preservation [6].

Polyphenols are abundantly found in the human diet, particularly in fruits and vegetables and derived products such as juices. In 2018, consumption of fruit juice and nectar in Europe was 9.2 billion liters, with orange being the preferred flavor (36.5%), followed by other fruits (21.8%), mixed flavors (19.2%), and apple (15.7%) [7]. Epidemiological studies have shown that fruit and vegetable juice intake may have a cardio-protective effect, but only a small impact on cancer development [8]. Moreover, interventional studies (focusing on acute or chronic conditions) with different juices (apple, orange, mandarin, cranberry grape, pomegranate) have evidenced decreased inflammation, arterial blood pressure, and plasma levels in some oxidative stress biomarkers associated with increased cardiovascular risk (lipid peroxides, oxidized LDL, carbonyl groups) [9,10,11,12].

The food industry has largely relied on antioxidant properties to enhance the health effects of its products. The popular in vitro ORAC (oxygen radical antioxidant capacity) assay, among several methods [13,14], has become the essential marketing argument for industrialists aiming to promote antioxidant properties of foods, and of fruits and vegetables juices in particular [15,16]. Yet the ORAC assay was very quickly criticized for many reasons: free radicals produced in vitro but having no physiological relevance, the absence of standardization, variable value expression, a great influence of the analytical procedure (making inter-laboratory comparisons of ORAC values impossible), etc. [17,18]. From a general point of view, the methods to determine the total in vitro antioxidant capacity of a food matric can be divided into two major groups: the methods based on single electron transfer reaction or SET (TEAC (trolox equivalent antioxidant capacity), FRAP (ferric reducing ability of plasma, CUPRAC (cupric ion reducing antioxidant capacity, and DPPH (2,2-diphenyl-1-picrylhydrazyl radical scavenging capacity) assays and the methods based on hydrogen atom transfer reaction or HAT (crocin bleaching assay, TRAP (total peroxyl radical trapping antioxidant parameter)) and assays. Advantages and disadvantages of all these methods have been discussed earlier [14]. However, antioxidant properties and their potential beneficial effects of phenolic compounds can be strongly affected by both their bioaccessibility (digestion and absorption efficiency) and bioavailability (ratio of active ingredient absorbed and detected in the target site to the total amount of orally ingested drug products) [19]. Caco-2 cell models and the Simulator of the Human Intestinal Microbial Ecosystem (SHIME^®^) are two in vitro models allowing both parameters to be determined [20]. Recently, in vivo laboratory methods using the *boulardii* strain have been used to evidence the correlation between the antioxidant activity of phenolic compounds present in high amounts in plants and their bioavailability index [21]. Using a rat model, Gerardi et al. [22] evidenced that the antioxidant capacity of plasma as measured by DPPH and FRAP assays was increased after intake of pomace products in a dose-response effect. Nevertheless, the use of both assays to evidence in vivo antioxidant activity largely remains a matter of debate [23]. In a more accurate way, Curti et al. [24] showed in a mouse model that oral administration of whole brown propolis extract containing galangin is followed by rapid absorption and metabolization of the latter, resulting in adaptations of the antioxidant enzymatic defense system.

To evaluate the antioxidant capacity of fruit and vegetable juices, it appears highly desirable to have an experimental assay in which a physiological radical is produced and/or that mimics in vivo conditions. Under in vivo conditions, increased superoxide anion free radical production (univalent reduction of oxygen) results from mitochondrial respiratory chain [25,26] and endothelial [27] dysfunctions, xanthine oxidase activation [28], and activation of cellular NADPH oxidases (NOX proteins, the prototype of which is the phagocyte NADPH oxidase) [29,30,31]. With whole blood samples and phorbol myristate acetate (PMA) as activator of NADPH oxidase activity, a respiratory burst occurs in white blood cells, leading to superoxide anion overproduction detectable by luminol-dependent chemiluminescence [32].

In a previous work, we investigated how both the total and individual polyphenol contents of 22 commercial fruit and vegetable juices might regulate the ex vivo vasorelaxation of aorta segments isolated from rats [33]. On the basis of these data, we set in the present study a new goal: to compare the antioxidant activities of fruit and vegetable juices as measured by the ORAC and chemiluminescence assays.

## 2. Materials and Methods

### 2.1. Materials

Cyanidin-3-*O*-glucoside (CyG), catechin (C), epicatechin (EC), epicatechin gallate (ECG), gallo catechin (GC), epigallocatechin (EGC), epigallocatechin gallate (EGCG), cyanidin-3-*O*-rutinoside (CyR), delpinidin-3-*O*-glucoside (DG), peonidin-3-*O*-glucoside (PG), malvidin (M), pelargonidin (Pel), peonidin (P), and petunidin (Pet) were purchased from Extrasynthese, Lyon, France. Kaempferol (Kaemp), myricetin (Myr), and quercetin (Quer) were obtained from Merck KGaA, Darmstadt, Germany. Phorbol 12-myristate 13-acetate (PMA), *N*,*N*′-Dimethyl-9,9′-biacridinium dinitrate (lucigenin), and fluorescein sodium salt were purchased from Sigma-Aldrich (St. Louis, MO, USA). 2,2′-Azobis(2-methylpropionamidine) dihydrochloride (AAPH), used as a peroxyl radical generator in the ORAC assay, was ordered from Fluka Chemie GmbH (Buchs, Switzerland).

Twenty-one commercial fruit and vegetable juices were selected from various Belgian and French supermarkets: (1) tomato (Carrefour), (2) tomato (Biotta), (3) carrot (Biotta), (4) orange (Carrefour), (5) pure orange (Vitamont), (6) lemon (Bonneterre), (7) grapefruit (Carrefour), (8) pure grapefruit (Vitamon), (9) grape (Materne), (10) pure grape (Vitamont), (11) pomegranate (Biotta), (12) blackcurrant (Biotta), (13) blackcurrant (Zimmers), (14) blackcurrant (Jacoby bio), (15) blackcurrant (Van Nahmen), (16) blackcurrant (Gut & Günstig), (17) blackcurrant (Albi), (18) pineapple (Carrefour), (19) pineapple juice (De Drie Wilgen), (20) apple (Carrefour), (21) pure apple (Vitamont). All juices were kept at +4 °C until analysis, which was performed within two days after purchase. If necessary, juices were filtrated in order to remove pulp.

### 2.2. Methods

The total polyphenol content (TPC) in all filtrated juices was evaluated in our previous paper [33] using the Folin–Ciocalteu method [34] directly after opening the bottles (limit of quantification (LOQ): 2 mg gallic acid equivalent (GAE)/L, 0.9 ≤ *R*^2^ ≤ 1). The determinations of specific phenolic compound (anthocyanin, flavanol, and flavonol families) amounts were also described in the same publication [33].

#### 2.2.1. Inhibition of Superoxide Anion Production (Chemiluminescence Method)

The respiratory burst test was performed according to the protocol described by Baptista et al. [27] on heparin-treated whole blood containing red blood cells, white blood cells, and platelets. Briefly, a 200 µL blood sample from three subjects, 50 µL lucigenin (10^−3^ M), and 10 µL freshly and filtrated fruit or vegetable juice diluted 10 times in phosphate buffered saline (PBS, pH 7.4) were mixed and incubated for 15 min at 37 °C in microplates. After blood stimulation with PMA (4 × 10^−6^ M), resulting in increased superoxide anion production, the maximal fluorescence emitted over a 30 min period was measured in quadrupla with a GloMax^®^ Multi Microplate Multimode luminometer (Promega, Madison, WI, USA). The difference between areas under the curves obtained in the presence and absence of PMA was defined as 100% activation. The percentage of superoxide anion inhibition was determined by calculating the area under the curve in the presence of both PMA and a fruit or vegetable juice. The limit of quantification (LOQ) of the assay was determined as LOQ_SOD_ by plotting the inhibition percentage vs. superoxide dismutase (SOD) as a specific inhibitor of superoxide anion, with concentrations in the range of 0 to 1.25 units SOD/mL as final concentration. This allowed us to determine that a cut-off of ≥20% inhibition corresponded to a LOQ_SOD_ of 0.5 U/mL.

**Figure 1:**
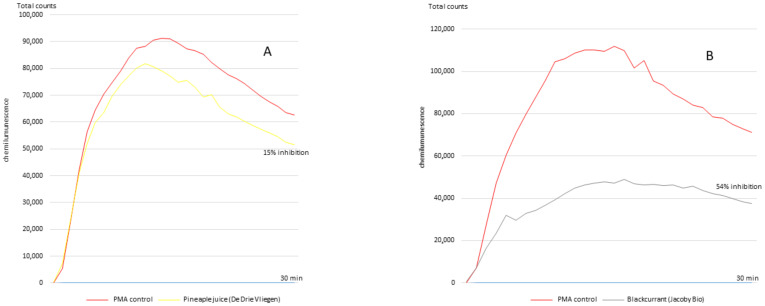
Examples of superoxide anion inhibition by pineapple (De Drie Wilgen) or blackcurrant (Jacoby bio) juice (panels (**A**) and (**B**), respectively) in PMA-activated whole human blood, as determined by chemiluminescence detection (30 min). The area under the curve for each analyzed juice yields the percentage of superoxide anion inhibition in relation to the PMA control.

Figure 1 shows typical chemiluminescence graphs recorded for juice 18 (pineapple, De Drie Wilgen, Nijlen, Begium) and juice 14 (blackcurrant, Jacobi Bio, Auggen, Germany). The protocol, consisting of drawing blood samples for antioxidant determination, was approved by the institutional ethics committee of Liège University Hospitals (B707201834834; reference 2017/342) and conducted in accordance with the 1964 Declaration of Helsinki and the European guidelines for good clinical practice.

#### 2.2.2. Oxygen Radical Antioxidant Capacity (ORAC Assay)

AAPH (2,2-azobis [2-amidinopropane] dihydrochloride) was used as a peroxyl radical generator and fluorescein as a fluorescent probe. Filters were used to select an excitation wavelength of 485 nm and an emission wavelength of 535 nm [18]. A total of 175 μL mixture containing fluorescein (3 μM), and AAPH (221 mM) was injected into each well of the microplate. All solutions were prepared in phosphate buffer 1M pH 7.4. Then 25 μL filtrated juice at an appropriate dilution, blank, or Trolox calibration solution (50–200 μM) were added. The fluorescence at 37 °C was recorded every 2 min for 1.5 h with a Victor3 multiplate recorder (Perkin Elmer, Zaventem, Belgium). The final ORAC value was calculated from the net area under the fluorescence decay curve. All assays were performed in triplicate. Results are expressed in micromoles of Trolox equivalents per liter (μM TE) (LOQ: 15 µM TE, 0.9 ≤ *R*^2^ ≤ 1).

### 2.3. Statistical Analyses

Correlations between ORAC values, TPC (expressed in µg GAE/mL), total and individual flavonol, flavanol, and anthocyanin concentrations, as well as percent superoxide anion inhibition, were calculated with Sisvar 5.6 software (University of Lavras, Lavras, Brazil). Pearson correlations were considered significant at *p* < 0.05.

## 3. Results

As shown in Table 1, already published in [28], the range of TPC values was wide. Juice 1 (tomato, Carrefour, Brussels, Belgium) showed the lowest value (214 µg GAE/mL) and juice 9 (Grape, Materne, Floreffe, Belgium), the highest (1564 µg GAE/mL). The mean TPC for the juices investigated was 852 ± 411 µg GAE/mL. As shown in a previous paper [28], the average flavonol and flavanol concentrations were, respectively, 6.3 ± 4.8 and 108 ± 254 µg/mL. Among the 21 tested juices, only the nine red ones (blackcurrant, grape, pomegranate) contained detectable levels of anthocyanins [28]. The mean total anthocyanin content was 131 ± 123 µg/mL for the nine red juices and 173 ± 132 µg/mL for the six blackcurrant juices considered alone.

Table 1 also shows that all blackcurrant and pomegranate juices caused more than 50% inhibition of superoxide anion production in PMA-activated whole blood. All the other juices showed superoxide anion scavenging activity in the range of 13.3 to 41.2%. The antioxidant profiles obtained with the ORAC assay were different: Only juices 8t (pure grapefruit, Vitamon, Monflanquain, France), 9 (grape, Materne, Floreffe, Belgium), 12 (blackcurrant, Biotta, Tägerwilen, Swiss), 17 (blackcurrant, Albi, Tournefeuille, France), and 21 (pure apple, Vitamont, Monflanquain, France) exhibited an ORAC value above 10,000 µM TE. A group of 13 juices [4,5,6,7,10,11,13,14,15,16,18,19,20], including the red ones, had ORAC values between 6074 (orange d’Espagne, Carrefour, Brussels, France) and 9259 (pure orange, Vitamont, Montflanquain, France) µM TE. Tomato and carrot juices exhibited the lowest values (below 4000 µM TE) of all the tested beverages. Data were strongly different when using the PMA-activated whole blood. For example, juice 9 (grape, Materne, Floreffe, Belgium) had the highest ORAC value (15,603 µM TE) but ranked eighth in terms of its ability to reduce superoxide anion production in the PMA–whole blood assay (41.2%). Conversely, juice 15 (blackcurrant, Van Nahmen, Hamminkeln, Germany) showed the greatest capacity to inhibit superoxide anion production (64.4%), but ranked 11th according to its ORAC value (7681 µM).

**Figure 2:**
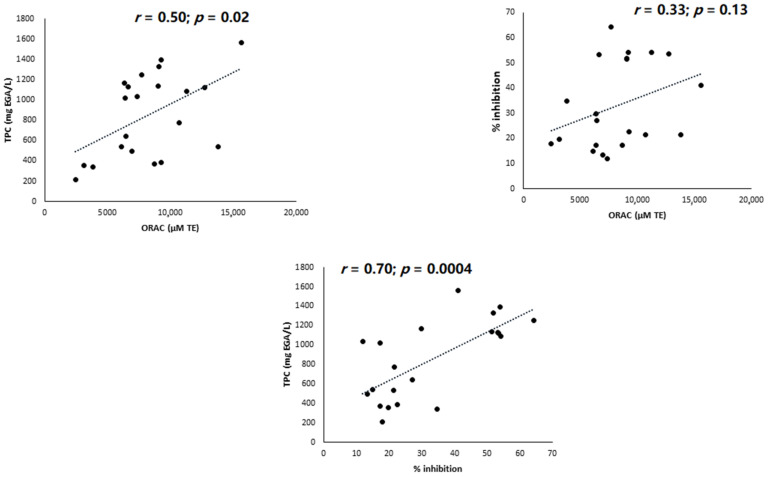
Graphic correlations between total polyphenol content (TPC), Oxygen Radical Antioxidant Capacity (ORAC) values, and % superoxide anion inhibition. EAG: equivalent gallic acid; TE: trolox equivalent.

Figure 2 depicts graphically the correlations found between the three assays. All beverages considered, a significant positive correlation was observed between TPC and the ORAC assay (*r* = 0.50, *p* = 0.02). A stronger and more significant correlation was observed between TPC and % superoxide anion inhibition (*r* = 0.70, *p* = 0.004). It is noteworthy that no correlation was observed between % superoxide anion inhibition and the ORAC assay (*r* = 0.33, *p* = 0.13)

Knowing the contents of all the juices in individual phenolic compounds of the flavonol, flavanol, and anthocyanin families (see our paper [28]) and the volume (10 µL) of 10-times-diluted juice added in the whole blood assay (270 µL), it was easy to calculate the exact mass concentration (µg/mL) of each phenolic compound in the reaction medium associated with the % superoxide anion inhibition, as established in Table 1. After the conversion of mass concentrations (µg/mL) to molar concentrations (µM) for each individual phenolic compound and taking into account the whole juices, Table 2 shows that the mean concentrations of Myr, Querc, Kaemp, PG, ECG, C, and EC (potentially associated with decreased superoxide anion production in PMA-stimulated whole blood) were low, ranging from 0.06 ± 0.072 (Myr) to 0.008 ± 0.014 (C) µM. Table 3 shows, for the whole set of juices, the correlations between concentrations of individual phenolic compounds on the one hand and either the ORAC value or the percentage of superoxide anion inhibition on the other. Only the Myr, ECG, and C concentrations were found to correlate positively and significantly with the ORAC values, whereas very strong correlations were observed between superoxide anion inhibition and PG (*r* = 0.87, *p* = 0.010), EGCG (*r* = 0.67, *p* = 0.0009), and, to a lesser extent, C (*r* = 0.47, *p* = 0.02) and Q (*r* = 0.46, *p* = 0.036).

## 4. Discussion

In order to claim health effects of fruit- and vegetable-derived products such as juices, the food industry has highlighted their antioxidant properties. To do so, manufacturers have relied on the first database of ORAC values published in 2007 by the United States of Department of Agriculture (USDA) [35], containing 277 selected foods at the time and extended to 326 foods in 2010 [36]. Despite the counterarguments of long-time ORAC researcher R. Prior, the USDA decided in 2012 to withdraw its web publication of ORAC values due to lack of scientific evidence that ORAC has in vivo relevance in terms of human health [37]. The USDA also mentioned that ORAC values were misused by food and dietary supplement companies as the sole basis for guiding consumer choices [37]. Even when a strong positive correlation was evidenced between ORAC values and the TPC of a food matrix, the European Food Safety Authority (EFSA) issued guidelines that forbade claiming, on food product labels, an antioxidant benefit on the basis of data obtained from any in vitro assay, including the TRAP, FRAP, TEAC, ORAC, and FOX assays [38,39]. Nevertheless, numerous food companies and marketers continue to promote products having the “highest” ORAC values. One major criticism formulated against the ORAC assay is that the generated free radicals result from the thermal decomposition of a purely chemical compound and that they therefore have no in vivo relevance. Furthermore, the fact that ORAC values are strongly affected by experimental conditions makes inter-laboratory comparisons impossible [18]. In the present paper we therefore suggest using an ex vivo blood test, in better agreement with physiological conditions, to test the antioxidant capacities of fruit and vegetable juices.

In vivo, the superoxide anion is the first physiological ROS resulting from the univalent reduction of oxygen. Among many possible in vivo sources of ROS, the NADPH oxidase family of enzymes (NOX1, NOX2, NOX4, NOX5) has been identified as an important one [29]. NOX2 is found notably in endothelial cells and adventitial fibroblasts, and also in phagocytic cells such as neutrophils (or polymorphonuclear leukocytes (PMNs)), macrophages, and monocytes. In resting PMNs, NOX2 is inactive. Upon exposure to stimuli, increased oxygen consumption (a respiratory burst) occurs in PMNs, leading to excess production of superoxide anion and hydrogen peroxide in the extracellular medium through activation of NOX2. There is strong evidence that excessive NOX2 oxidase activity in PMNs plays an important role in the development of vascular inflammation and cardiovascular disease, including hypertension (via inhibition of eNOS by superoxide anion), atherosclerosis, diabetes, cardiac hypertrophy, and heart failure [30,31]. As early as 1986, Pagonis et al. [40] described that some flavonoids, in their aglycone form (quercetin, kaempferol, morin), can limit ROS production by in vitro stimulated neutrophils. Possible mechanisms underlying ROS inhibition were attributed to NADPH oxidase inhibition through the blocking of phospholipase D (signaling pathway) [41] and, to a lesser extent, to direct superoxide anion scavenging [42]. Using luminol-dependent fluorescence, Zielinska et al. [43] evidenced that the intracellular production of ROS in stimulated neutrophils depends on the number of hydroxyl groups present on rings A and B of flavonoids, in agreement with other studies [44,45]. The antioxidant and anti-inflammatory activities of phenolic compounds, associated with protective effects against major chronic diseases such as cardiovascular disease, have been well described [46,47].

Phorbol myristate acetate, such as protein kinase C agonists, is well known to induce NADPH oxidase activation in neutrophils. In both isolated PMNs and whole blood, different detection techniques (fluorescence, chemiluminescence, accumulation of a dye in cells, electron spin resonance spectroscopy) have been used to evidence superoxide anion production. In the present study, we used the protocol described by Baptista et al. [32] involving PMA stimulation in whole blood and the detection of superoxide anion by chemiluminescence, involving an in-house-automated process using 96-well microplates introduced into a luminometer. Using this method to measure the antioxidant activities of polyphenol-rich fruit and vegetable juices, we achieved results within 30 min of the start of the test, as opposed to two hours with the ORAC assay. In contrast to this last test, Table 1 clearly highlights a common and powerful antioxidant capacity for all blackcurrant juices when using the PMA–whole blood assay. Figure 2 also shows that with this test, a greater and more significant correlation was observed (*r* = 0.70, *p* = 0.0004) between TPC and % superoxide anion inhibition than between TPC and the ORAC test (*r* = 0.50, *p* = 0.02). Of interest is the absence of correlation between the ORAC and PMA–whole blood assays, demonstrating the higher sensitivity of the latter in determining the antioxidant capacities of fruit and vegetable juices.

Unlike the ORAC assay, this ex vivo biological test on blood could allow the potential in vivo relevance of the presence of specific phenolic compounds in juice to be evaluated. Although the polyphenols of most classes in their aglycone forms are sufficiently absorbed through the intestinal barrier, their concentrations in the bloodstream reach only 1 µM, in contrast to other antioxidants such as glutathione (600 µM), vitamin C (60 µM), and vitamin E (10 µM). Manach et al. [48] showed the catechin (C) concentration in human plasma to range from 0.14 to 0.49 µM after ingestion of 0.36 mg/kg pure C. After consumption of flavonol at 110 mg/day for 14 days, the quercetin concentration in human plasma was found to increase from 0.078 µM, observed with a baseline diet, to 0.304 µM [49]. Liu et al. [50] found the plasma concentration of cyanidin-3-*O*-galactoside to peak at 0.0031 µM about two hours after ingestion of 100 g Saskatoon berries containing 123.5 mg of this compound. Giordano et al. [51] reported a plasma cyanidin-3-*O*-glucoside (CyG) level reaching 0.0040 µM one hour after ingestion of 500 mL orange juice containing 12.89 mg CyG. Rechner et al. [52] observed peak plasma concentrations of delphinidin-3-*O*-glucoside (0.006 µM), delphinidin-3-*O*-rutinoside (0.051 µM), cyanidin-3-*O*-glucoside (0.0035 µM), and cyanidin-3-*O*-rutinoside (0.024 µM) about one hour after ingestion of 330 mL blackcurrant juice concentrate containing 1 g total anthocyanins.

In our previous study [33], we determined in each fruit and vegetable juice tested here the precise amounts of phenolic compounds belonging to the flavonol, flavanol, and anthocyanin families. Table 2 shows that, all juices considered, the majority of phenolic compounds liable to inhibit superoxide anion production did so at mean concentrations in the physiological range (<1 µM). PG, an anthocyanin present in blackcurrant juices, appeared particularly effective, with a mean value as low as 0.005 µM. Table 3 shows that only PG, EGCG, C, and Q could be selected as potential in vivo-acting candidates, thanks to their strong and positive correlations with superoxide anion inhibition at potential in vivo concentration. According to previous studies [43,44], PG, Q, C, and EGCG are likely to exert superoxide anion scavenging activity because they are characterized by the presence of additional –OH groups on ring B. Our observations were, however, opposite to experiments on stimulated human neutrophils showing that pure flavonoids such as quercetin had to be present at levels well above their physiological concentrations, i.e., in the range 1 mM (22.4 % inhibition) to 100 mM (90.7% inhibition), in order to exert “antioxidative” activity [43]. Possibly, therefore, the reduction in superoxide anion production by fruit juices in the blood test might result from synergy between all the phenolic compounds present in the beverages. Mechanistically, recent studies have also highlighted that the protective effects of dietary polyphenols can be attributed to hormetic pathways rather than to a direct antioxidant effect. In cells, polyphenols at low concentration may produce, through moderate autooxidation, small amounts of ROS capable of inducing, via activation of the Keap1/Nrf2/ARE pathway, the expression of gene-encoding antioxidant enzymes (antioxidant response element) and phase-2 enzymes [53,54,55].

### Limitations of the PMA–Whole Blood Assay

Although it yields more precise information than the ORAC assay regarding the potential antioxidant capacities of fruit and vegetable juices and of their phenolic compounds in particular, the PMA–whole blood assay has limitations. Firstly, it is an invasive method requiring a blood test. Secondly, it does not take into account factors such as bioavailability, metabolism, or synergistic or antagonistic effects of phenolic compounds present in the beverages. The forms appearing in the blood are different from those found in foods. The only way to evaluate scientifically the antioxidant capacity of a given polyphenol-rich beverage is to conduct an in vivo human study. Subjects would be asked to consume a determined volume of juice with a well-characterized content of phenolic compounds. One to four hours later, the plasma concentrations of those compounds (and/or of their metabolites, if possible) would be measured and compared with pre-ingestion values (control). Then one could use the ex vivo PMA–whole blood assay, with concentrations of pure phenolic compounds similar to those detected in vivo, to get a better picture of potential in vivo “antioxidant” properties of fruit and vegetable juices.

## 5. Conclusions

Although used abundantly by food companies to support claims regarding health benefits of products such as fruit juices, the ORAC scores reflecting in vitro antioxidant activity are not adequate for this purpose. This is because this assay uses a non-physiological ROS, is greatly influenced by experimental conditions, and has no in vivo relevance recognized by food safety agencies. It is thus imperative to use more appropriate assays based on biological materials. An example is the ex vivo PMA–whole blood assay, which allows evaluating how some given juice rich in antioxidants, particularly polyphenols, interacts with the superoxide anion, i.e., the first physiological radical produced by univalent reduction of oxygen. Assessing the potential in vivo relevance of this assay, however, will require performing it in parallel with precise determinations of all phenolic compounds present in fruit juices.

## Figures and Tables

**Table 1:** Total polyphenol content (TPC) [28], Oxygen Radical Capacity (ORAC) values, and % of superoxide anion inhibition by PMA-activated-whole blood assay of 21 commercial fruit and vegetables juices (*n* = 3 for TPC and ORAC assay; *n* = 4 for % superoxide anion inhibition). Italic numbers into brackets indicate the ranking from highest to lowest antioxidant capacity of all juices according to the test used.

Number	List of Juices	TPC	ORAC (µM TE)	% Superoxide Anion Inhibition
		(mg GAE/L)		(PMA-Whole Blood)
1	Tomato (Carrefour)	213.6 ± 68.1	2428.7 ± 68.2 *(21)*	18.0 ± 5.9 *(16)*
2	Tomato (Biotta)	358.1 ± 26.0	3117.8 ± 290.1 *(20)*	19.8 ± 19.0 *(15)*
3	Carrot (Biotta)	338.9 ± 5.6	3825.9 ± 840.0 *(19)*	34.7 ± 6.5 *(9)*
4	Orange d’Espagne (Carrefour)	541.6 ± 90.1	6074.3 ± 745.2 *(18)*	14.9 ± 4.6 *(19)*
5	Pure orange (Vitamont)	385.1 ± 155.8	9259.3 ± 288.2 *(6)*	22.6 ± 7.7 *(12)*
6	Lemon (Bonneterre)	1167.5 ± 217.3	6346.3 ± 628.6 *(17)*	29.8 ± 17.4 *(10)*
7	Grapefruit	496.8 ± 13.1	6936.5 ± 1482.1 *(13)*	13.3 ± 3.7 *(20)*
8	Pure grapefruit (Vitamont)	537.6 ± 45.8	13,805.2 ± 591.3 *(2)*	21.5 ± 9.1 *(14)*
9	Grape (Materne)	1564.0 ± 588.3	15,603.6 ± 458.5 *(1*)	41.2 ± 3.3 *(8)*
10	Pure grape (Vitamont)	643.4 ± 56.6	6461.7 ± 808.7 *(15)*	27.1 ± 2.7 *(11)*
11	Pomegranate (Biotta)	1331.9 ± 183.2	9046.1 ± 176.8 *(8)*	51.9 ± 5.4 *(6)*
12	Blackcurrant (Biotta)	1088.8 ± 80.8	11,256.7 ± 380.1 *(4)*	54.3 ± 7.4 *(2)*
13	Blackcurrant (Zimmers)	1392.2 ± 202.7	9219.4 ± 466.0 *(7)*	54.1 ± 4.9 *(3)*
14	Blackcurrant (Jacoby Bio)	1135.6 ± 93.4	9035.1 ± 90.6 *(9)*	51.5 ± 2.5 *(7)*
15	Blackcurrant (Van Nahmen)	1250.9 ± 186.8	7681.7 ± 422.9 *(11)*	64.4 ± 12.4 *(1)*
16	Blackcurrant (Gut & Günstig)	1131.1 ± 210.8	6636.8 ± 577.5 *(14)*	53.2 ± 8.5 *(5)*
17	Blackcurrant (Albi)	1121.6 ± 166.2	12,722.9 ± 622.4 *(3)*	53.5 ± 6.9 *(4)*
18	Pineapple juice (Carrefour)	1018.6 ± 60.8	6363.1 ± 1141.8 *(16)*	17.2 ± 4.5 *(17)*
19	Pineapple juice (De Drie Wilgen)	369.6 ± 21.6	8700.1 ± 213.6 *(10)*	17.2 ± 3.4 (*18)*
20	Apple (Carrefour)	1035.3 ± 8.7	7351.3 ± 2924.7 (*12)*	11.9 ± 6.8 *(21)*
21	Pure apple (Vitamont)	776.4 ± 121.0	10,682.4 ± 1965.4 *(5)*	21.6 ± 2.8 (*13)*

**Table 2:** Mean concentrations (µM), established for the whole set of juices, of phenolic compounds present in the PMA–whole blood assay (260 µL) associated with inhibition of superoxide anion production.

	**Flavonols (µM)**
Myr	0.060 ± 0.072
Quer	0.039 ± 0.019
Kaemp	0.011 ± 0.009
	**Flavanols (µM)**
EGC	0.949 ± 3.266
EGCG	0.332 ± 0.472
ECG	0.043 ± 0.040
C	0.008 ± 0.014
ECG	0.043 ± 0.033
	**Anthocyanins (µM)**
DG	0.745 ± 1.531
DR	1.633 ± 3.540
CyG	0.196 ± 0.308
CyR	1.424 ± 2.720
PG	0.005 ± 0.005
GC	0.367 ± 1.141

**Table 3:** Correlations, established for the whole set of juices, between individual phenolic compounds present in the PMA–whole blood assay and either the ORAC values or the % inhibition of superoxide anion production.

Phenolic Compounds	ORAC		% Inhibition	
	*r*	*p*-Value	*r*	*p*-Value
DG	0.32	0.40	0.17	0.66
DR	0.37	0.32	0.19	0.63
CyG	0.36	0.34	0.22	0.57
CyR	0.29	0.44	0.18	0.65
PG	0.25	0.52	0.87	0.010
GC	0.04	0.87	0.21	0.33
EGC	0.11	0.62	0.28	0.20
EGCG	0.30	0.19	0.67	0.0009
ECG	0.45	0.043	0.26	0.24
C	0.47	0.032	0.47	0.02
EC	0.28	0.21	0.04	0.99
Myr	0.48	0.027	0.33	0.14
Quer	0.21	0.36	0.46	0.036
Kaemp	0.13	0.57	0.24	0.30

## Data Availability

The datasets analyzed during the current study are available from the corresponding author on reasonable request.
